# Limbal Approach Phacovitrectomy to Treat Cataract with Clinically Significant Asteroid Hyalosis—Presentation of the Technique and Preliminary Results

**DOI:** 10.3390/jcm10153338

**Published:** 2021-07-28

**Authors:** Agnieszka Rozegnał-Madej, Aleksandra Wlaź, Tomasz Żarnowski

**Affiliations:** Department of Diagnostics and Microsurgery of Glaucoma, Medical University, 20-079 Lublin, Poland; aleksandra.wlaz@icloud.com (A.W.); zarnowskit@poczta.onet.pl (T.Ż.)

**Keywords:** cataract, asteroid hyalosis, phacovitrectomy, new surgical technique

## Abstract

Purpose: To assess preliminarily the efficacy and safety of a relatively new surgical modification of phacovitrectomy in eyes with cataract and visually significant asteroid hyalosis (AH). Materials and methods: Prospective, noncomparative, interventional case series of six eyes of six patients (mean age 75.6 years; 1 woman, 5 men) with cataract and visually significant AH treated with a novel surgical technique—a phacoemulsification with anterior vitrectomy through posterior capsulorhexis and intraocular lens (IOL) implantation. Main outcome measures were: best-corrected visual acuity (BCVA), intraocular pressure (IOP), IOL centration, complications. Mean follow-up was 39.17 ± 4.31 months. Results: The mean BCVA (Snellen) improved from 0.26 ± 0.18 to 0.73 ± 0.33 at the end of the follow-up. IOP was in the normal range, and no problems with IOL fixation were observed at the end of the follow-up. No post-operative complications, retinal detachment, retinal tears, macular edema or prolonged inflammation were observed. Conclusions: The presented new surgical technique seems to be a safe and efficacious method to treat cataract with visually significant asteroid hyalosis.

## 1. Introduction

Asteroid hyalosis (AH), first described by Benson in 1894, is a degenerative condition in which small yellow–white plaques called asteroid bodies (ABs) are suspended in the vitreous body, moving along with the movements of the eye or head [1]. This rare condition affects 0.8% to 1.2% of the population [2,3] and is primarily associated with aging. AH is predominantly unilateral and more prevalent in men [3,4,5].

Cataracts, like AH, mainly affect elderly patients. Many of them, despite perfect cataract surgery, are not satisfied because of troublesome vitreous floaters. To meet their expectations, pars plana vitrectomy (PPV) is recommended as a separate standard procedure. We present a relatively new method combining cataract surgery and a removal of a significant bulk of ABs from the visual axis. This approach is fast, simple and may be performed simultaneously with cataract surgery by an anterior segment surgeon.

The aim of the present study is to evaluate the safety and efficacy of the surgical technique that consists of phacoemulsification followed by an anterior vitrectomy performed through a posterior capsulorhexis.

## 2. Materials and Methods

Six eyes of six patients were treated in the Department of Diagnostics and Microsurgery of Glaucoma of Medical University in Lublin, Poland between April 2016 and February 2017. The protocol of this study was approved by the Bioethics Committee of Medical University in Lublin (Poland) and registered under the number KE-0254/110/2020. The aims of the study, the benefits and risks of the procedure were explained thoroughly to the patients and written informed consent was obtained from all the participants in accordance with tenets of the Declaration of Helsinki.

The inclusion criteria were cataracts with visually significant AH and best corrected visual acuity (BCVA) ≤ 0.6 (Snellen decimal units), with PVD and normal peripheral retina. Only patients with symptomatic AH with complaints of glare and floaters were involved in the study. The exclusion criteria were no PVD, macular hole or epiretinal membrane, any peripheral retina pathologies, history of retinal detachment or intraocular inflammation.

All patients underwent a baseline ophthalmic examination with slit-lamp biomicroscopy, indirect ophthalmoscopy with very careful retina assessment, BCVA and IOP measurement, optical coherence tomography (OCT; Cirrus HD-OCT V.7.0.3.19, Carl Zeiss Meditec Inc., Dublin, CA, USA) and ultrasound B scans (USG; Aviso A/B, V.4.0.2, Quantel Medical, Rockwall, TX, USA) on each visit. Main outcome measures were BCVA, IOP, intraocular lens (IOL) centration and possible complications. Examinations were performed before and after the surgical intervention. Follow-up visits were arranged at 7 days, 30 days, 12 months and 36 months postoperatively.

### 2.1. Surgical Technique

**Figure 1:**
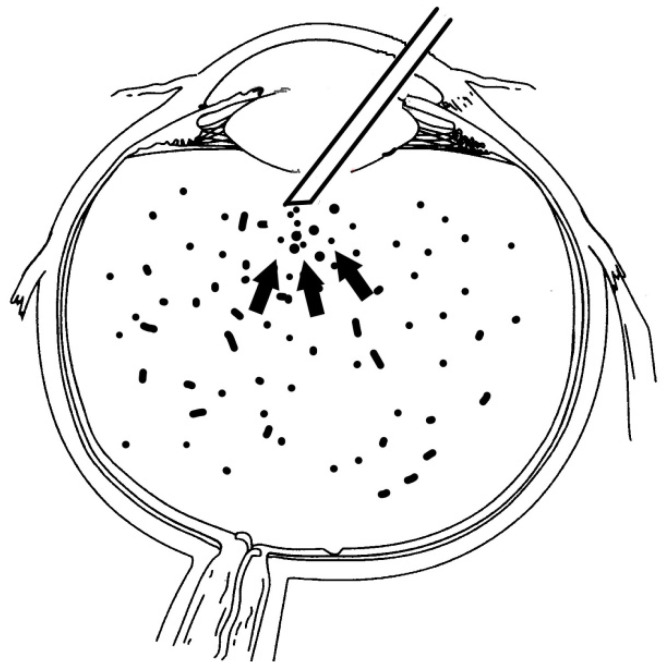
Diagram of the vitreous cutter placement during surgery.

**Figure 2:**
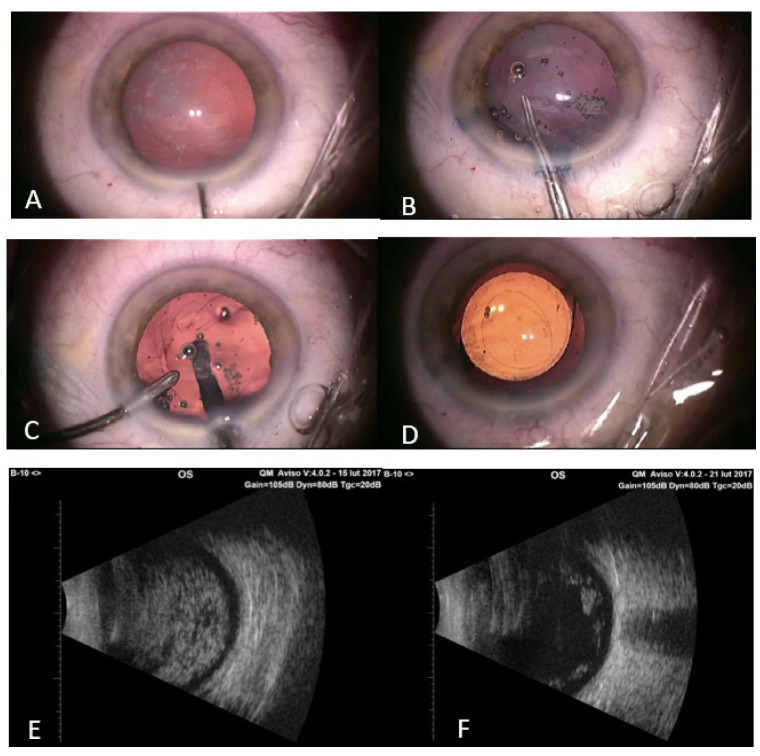
(**A**) Patient’s eye after phacoemulsification. (**B**) Posterior capsulorhexis with capsulorhexis forceps. (**C**) Twenty-gauge vitreous cutter introduced to the vitreous cavity behind the posterior capsule, AC maintainer introduced through the sideport to the anterior chamber. (**D**) Final result after PCIOL implantation. (**E**) Patient’s ultrasound B scan before surgery. (**F**) The same patient’s ultrasound B scan after surgery.

All surgeries were performed by the same anterior segment surgeon (TŻ) with peribulbar anaesthesia. After standard cataract removal by phacoemulsification, the posterior capsular bag was stained with trypan blue to improve visualisation. Subsequently, the dye was washed out with BSS. The capsular bag was inflated with a viscoealastic agent, and a small central tear was made in the posterior capsule with 25-gauge needle. Afterwards, small amount of viscoelastic agent was injected through the tear to tamponade the vitreous. A round 3.0–4.0 mm posterior continuous curvilinear capsulorhexis (PCCC) was performed using the capsulorhexis forceps. The posterior bag was grasped and re-grasped frequently to keep the opening central and smaller than the anterior capsulorhexis. A twenty-gauge vitreous cutter (INFINITI Vision Systems, Alcon Laboratories, Inc., Fort Worth, TX, USA) was introduced through newly created opening to the vitreous cavity with the concomitant infusion line as an anterior chamber (AC) maintainer. Central anterior cortical vitrectomy, about 3 mm deep, was performed to remove asteroid hyalosis from the central visual axis. The vitreous cutter was placed in the central vitreous cavity only, facing down, just behind the posterior capsule, to avoid tractions in the peripheral retina (Figure 1). Vitrectomy parameters were cut at the rate 600–800 cuts/min; vacuum 150–200 mmHg. The vitreous cutter was kept in the area of the PCCC and was never placed under the peripheral posterior capsule. Thereafter, AC was repeatedly filled with viscoelastic and posterior chamber acrylic IOL (Acrysof IQ SN60WF, Alcon Laboratories Inc., Fort Worth, TX, USA) was inserted into the capsular bag. Remaining viscoelastic agent was removed by irrigation and aspiration. Aprokam (cefuroxime, Thea Pharmaceuticals, Clermont-Ferrand, France) was injected into the AC at the end of the surgery (Figure 2). No vitreous prolapse was observed during or after the surgery in any of the presented cases.

### 2.2. Statistical Analysis

The statistical analysis was performed using GraphPad Prism version 5.03 (GraphPad Software, San Diego, CA, USA). Comparisons of continuous variables were performed using the Wilcoxon signed rank test. A repeated measures analysis of variance (ANOVA) with the Dunnett’s post-hoc test was done to test BCVA in function of time. Visual acuity in decimal notation was converted to LogMAR values before statistical analysis and then converted back to Snellen decimal. Normality of data was assessed using the Shapiro–Wilk test. Differences were considered statistically significant at *p* < 0.05.

## 3. Results

Six eyes of six patients underwent phacoemulsification with anterior vitrectomy through PCCC and IOL implantation. The follow-up was at least 36 months (39.17 ± 4.31). The mean age was 75.67 ± 2.50 years. The subjects comprised five males and one female. The patients’ demographics are shown in Table 1.

**Figure 3:**
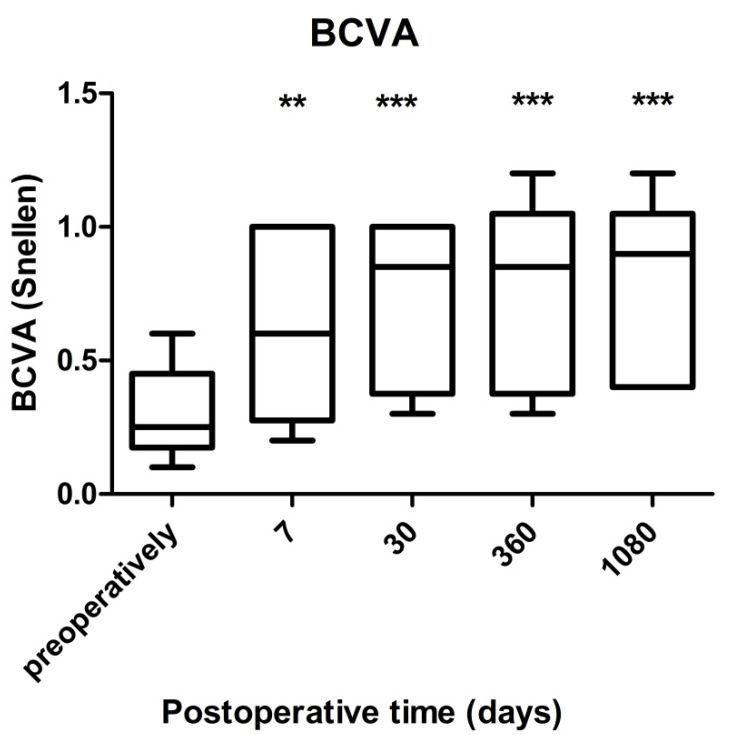
Box plot presentation of visual acuity at all follow-up visits in the study. Median values (horizontal lines), 25/75 percentiles (boxes), and minimum-maximum (whiskers). ANOVA: F(4, 5) = 20.32; *p* < 0.0001. A post hoc Dunnett’s test revealed statistical improvement in visual acuity from the respective baseline at each time point (** *p* < 0.01; *** *p* < 0.001).

The mean BCVA statistically significantly improved from 0.26 ± 0.18 to 0.73 ± 0.33 at the end of the follow-up (*p* = 0.0355, Wilcoxon signed-rank test) (Table 2). For 3 years after the surgery, there was significant change in visual acuity (ANOVA: F(4, 5) = 20.32; *p* < 0.0001) (Figure 3).

Three patients had a limited vision improvement as a consequence of coexisting glaucoma (patients 1, 2 and 6). We observed no changes in mean IOP before and after surgery (17.50 ± 2.17 and 14.00 ± 2.83, respectively; *p* = 0.0731, Wilcoxon signed-rank test).

All eyes had central IOL fixation without displacement. One of the essential points of our study was retina evaluation after surgery. No postoperative complications throughout the entire observation period were observed. We found no retinal detachment, no retina holes, no macular edema and no prolonged inflammation.

## 4. Discussion

Most AH cases are asymptomatic with unaffected vision even in severe conditions with dense opacities, which make fundus examination difficult or simply impossible. The reason for this is probably smooth surfaces of asteroid bodies which do not scatter light as disturbing fashion as other vitreous opacities [6]. However, posterior vitreous detachment (PVD) may cause ABs to be displaced anteriorly and accumulate directly behind the lens, which could markedly affect vision [5].

One of the possible treatments modalities used in symptomatic floaters is a neodymium:YAG (Nd:YAG) laser vitreolysis. Delaney et al. [7] estimate this procedure as safe but only moderately efficacious with an improvement for vitreous floaters in 38.3% of eyes. Coexisting cataract may complicate floaters’ visualization and disrupt dense opacities close to the retina can cause retinal damage. Therefore, laser vitreolysis is not recommended for treatment of symptomatic cataract with AH.

Pars planavitrectomy (PPV) combined with cataract surgery may be a good option in advanced cases. However, vitreous of patients with AH shows reduced gel liquefaction and anomalous vitreoretinal adhesions which can lead to complications during vitrectomy. In most cases intravitreal administration of triamcinolone acetonide is necessary to complete residual vitreous cortex removal [8]. These adhesions probably enhance with coexisting maculopathy or proliferative diabetic retinopathy. It has been reported that there is an increase in inflammation-related cells, such as macrophages, in the vitreous of AH and that prolonged mild inflammation might accelerate the manifestation of the adhesion molecules at the vitreoretinal interface [9].

Kitagaki et al. [9] presented two cases of idiopathic macular hole with AH. They performed standard phacovitrectomy and observed very strong vitreoretinal adhesion starting at the mid-periphery, resulting in multiple iatrogenic tears during surgery. As a conclusion, the authors determined that artificial PVD should be limited to the area of loose adhesion. The advantage of PPV is the ability to treat iatrogenic tears or other retinal pathologies immediately during surgery. Therefore, PPV is the first-choice method in cases with AH and cataract coexisting with retina pathologies.

To the best of our knowledge, combined phacoemulsification and deep anterior vitrectomy was previously introduced for floaters treatment by Mossa et al. [10]. In this report, 2 of 10 eyes developed postoperative cystoid macular edema and 1 eye had a recurrence of floaters. However, only three eyes were diagnosed with AH and follow-up was only 6 months. The authors implanted silicone IOLs (SI-40, Allergan) and in the current state of knowledge, silicon lenses are contraindicated due to calcification on IOL surface in eyes with AH [5].

In our case series, all six eyes were treated successfully using phacoemulsification combined with anterior vitrectomy through posterior capsulorhexis. As mentioned above, no complications were observed during an at least 36-month observation period. However, it is important to remember to follow certain rules that determine the effectiveness and safety of this procedure. The right preoperative assessment and careful ophthalmoscopy (to exclude retinal tears and confirm PVD) are crucial. However, peripheral retinal examination may be difficult due to dense asteroid hyalosis, which can be limitation of this surgical technique. Anomalous spontaneous PVD in AH may lead by itself to vitreoschisis, macular hole or epiretinal membrane formation. Lack of concomitant macular disease or diabetic retinopathy probably causes less strong vitreoretinal adhesion and a lower risk of complications. Posterior capsulorhexis is the most difficult stage that requires a learning curve. Central placement of the vitreous cutter in the vitreous cavity is the key point of success. We conclude that age-related liquefaction of the vitreous just behind the lens is enough to safely clean the visual axis and significantly improve the quality of vision of our patients. Sideways and deep movements of the cutting tip should be avoided to prevent pulling of the retina periphery and iatrogenic retinal damage. Finally, it is worth noting that the procedure performed simultaneously reduces costs, is less time-consuming, and does not expose the patient to stress associated with additional surgery in cases where pars plana vitrectomy is planned due to visually significant AH. The lack of the posterior capsule opacification, as a late complication of cataract surgery, is an additional benefit of this surgical technique.

The strength of the current study is long follow-up allowing a sufficient assessment of safety profile of the new technique. The major limitations of our study include a small sample size, heterogeneous study group and the lack of other than BCVA outcome measures. However, the main purpose of this study was to assess the safety profile of a new surgical technique and only patients with visually significant AH with disturbing symptoms were involved in the study. To minimize the risk of multiple surgeries, these patients were chosen to undergo one-step procedure during standard phacoemulsification cataract surgery.

## 5. Conclusions

Phacoemulsification with anterior vitrectomy through posterior capsulorhexis is a safe and efficient procedure for selected cases of cataract concomitant with visually significant AH. This technique is fast, relatively easy and possible to perform by anterior segment surgeons, who want to improve the quality of life of their patients with visually significant AH. No need to involve a vitreoretinal surgeon and planning another expensive procedure seems to be an attractive alternative for selected patients. The key to success is a proper qualification of the patients and careful core vitrectomy. However, comparative studies on larger groups of patients are needed to establish the long-term safety and efficacy of this surgical technique.

## Figures and Tables

**Table 1:** Summary of the patients’ data.

Patien tnumber	Demographic Data				BCVA (Snellen Decimal)				
	Age/Gender	Eye	Ocular History	Observation Time (Months)	Pre-op	7 Days	30 Days	12 Months	36 Months
1	73 ♂	L	PAOG	36	0.2	0.3	0.4	0.4	0.4
2	78 ♂	L	PAOG	43	0.2	0.3	0.3	0.3	0.4
3	75 ♂	L	no	46	0.3	1.0	1.0	1.2	1.2
4	76 ♂	R	no	36	0.4	1.0	1.0	1.0	1.0
5	79 ♂	L	no	36	0.6	0.9	1.0	1.0	1.0
6	73 ♀	R	PACG	38	0.1	0.2	0.7	0.7	0.8

Abbreviations: BCVA (Snellen decimal units), best corrected distance visual acuity; PACG, primary angle closure glaucoma; POAG, primary open angle glaucoma; R/L right/left eye.

**Table 2:** Visual acuity changes before the surgery and at the end of the follow-up.

BCVA Preoperatively (±SD) (Snellen Decimal)	BCVA 3 Years Postoperatively (±SD) (Snellen Decimal)	*p-* Value (Wilcoxon Signed-Rank Test)
0.26 ± 0.18	0.73 ± 0.33	0.0355

## Data Availability

The data presented in this study are available on request from the corresponding author. The data are not publicly available due to ethical restrictions and General Protection Data Regulation.
